# Ephrin-B2 expression critically influences Nipah virus infection independent of its cytoplasmic tail

**DOI:** 10.1186/1743-422X-5-163

**Published:** 2008-12-24

**Authors:** Lena Thiel, Sandra Diederich, Stephanie Erbar, Dennis Pfaff, Hellmut G Augustin, Andrea Maisner

**Affiliations:** 1 Institute of Virology, Philipps University of Marburg, Marburg, Germany; 2 Joint Research Division Vascular Biology, Medical Faculty Mannheim, University of Heidelberg (CBTM), and German Cancer Research Center (DKFZ), Heidelberg, Germany

## Abstract

**Background:**

Cell entry and cell-to-cell spread of the highly pathogenic Nipah virus (NiV) requires binding of the NiV G protein to cellular ephrin receptors and subsequent NiV F-mediated fusion. Since expression levels of the main NiV entry receptor ephrin-B2 (EB2) are highly regulated *in vivo *to fulfill the physiological functions in axon guidance and angiogenesis, the goal of this study was to determine if changes in the EB2 expression influence NiV infection.

**Results:**

Surprisingly, transfection of increasing EB2 plasmid concentrations reduced cell-to-cell fusion both in cells expressing the NiV glycoproteins and in cells infected with NiV. This effect was attributed to the downregulation of the NiV glycoproteins from the cell surface. In addition to the influence on cell-to-cell fusion, increased EB2 expression significantly reduced the total amount of NiV-infected cells, thus interfered with virus entry. To determine if the negative effect of elevated EB2 expression on virus entry is a result of an increased EB2 signaling, receptor function of a tail-truncated and therefore signaling-defective ΔcEB2 was tested. Interestingly, ΔcEB2 fully functioned as NiV entry and fusion receptor, and overexpression also interfered with virus replication.

**Conclusion:**

Our findings clearly show that EB2 signaling does not account for the striking negative impact of elevated receptor expression on NiV infection, but rather that the ratio between the NiV envelope glycoproteins and surface receptors critically influence cell-to-cell fusion and virus entry.

## Background

Nipah virus (NiV) was isolated in 1999 after an outbreak of severe respiratory illness in pigs and fatal encephalitis among pig farmers in Malaysia and Singapore [1,2]. Together with the closely related Hendra virus (HeV), NiV forms the new genus henipavirus within the *Paramyxoviridae *family [3,4]. With their exceptional wide host range, their zoonotic potential and their ability to cause fatal diseases in animals and humans, henipaviruses differ from all other known paramyxoviruses and are classified as Biosafety Level 4 (BSL4) pathogens. Fruit bats of the genus *Pteropus *have been identified as natural NiV reservoir. Besides bats, many other mammalian species such as pigs, horses, dogs, cats or humans can be infected [5-8]. During the first outbreak beginning in 1998, NiV was transmitted from bats to pigs, and then to humans. In more recent outbreaks in Bangladesh which were characterized by higher case fatality rates near 70% and rare human-to-human transmissions, there was no link to infected livestock or domestic animals. Here, NiV was likely transmitted to humans by date palm sap contaminated by bat secretions [9-11].

Infection of endothelial cells is a hallmark of NiV infection in animals and humans. Significant involvement of blood vessels in the central nervous system (CNS), lung, heart and kidney was observed in all infections. In humans, the severe damage of the microvasculature of the CNS is thought to be the basis for the development of the NiV encephalitis which often leads to coma and death within three to thirty days [12,13]. Typically, small arteries, arterioles, capillaries and venules in the brain showed evidence of vasculitis and thrombosis with frequent adjacent necrosis and hemorrhage. Syncytial or multinucleated giant endothelial cells were seen in blood vessels of various organs, and viral inclusions were found in endothelial cells as well as in brain parenchymal cells and neurons near vasculitic vessels or necrotic plaques [13]. As extensive viral replication in the CNS is assumed to be an important factor for high mortality [14], efficient NiV entry and spread from infected cells in the brain is likely crucial for the outcome of infection.

Successful NiV entry into host cells requires the concerted action of the two viral envelope glycoproteins F and G. After binding of the attachment protein G to suitable receptors on the cell surface, the fusion protein F in cooperation with the G protein promotes fusion of the viral envelope and the plasma membrane leading to virus entry. As with most paramyxoviruses, virus entry occurs at the cell surface and does not require receptor-mediated endocytosis [15]. After productive NiV replication, newly synthesized F and G proteins are expressed on the surface of the infected cell, and can trigger cell-to-cell fusion with receptor-bearing neighboring cells resulting in the formation of multinucleated syncytia [16]. To fulfill its function in fusion promotion during virus entry and cell-to-cell fusion, the NiV F protein must be proteolytically activated by cellular cathepsin L within an acidic endosomal compartment, before it is expressed on the cell surface and is incorporated into cell-free virus particles [15,17-19].

Ephrin-B2 (EB2) is known to act as main entry receptor for NiV [20,21], and its expression on endothelial cells, smooth muscle cells and neurons [22-26] is highly consistent with the known tropism of NiV infection *in vivo *[13]. Besides EB2, ephrin-B3 can function as alternate receptor and is likely used in brain regions where EB2 is not expressed [27,28]. EB2 is a transmembrane-anchored ligand of the receptor tyrosine kinases EphB2, EphB3 and EphB4. Interactions of Eph receptors with EB2 can trigger a wide array of cellular responses including cell adhesion, boundary formation and repulsion, and thus play a critical role in embryonic patterning, axon guidance, blood vessel remodeling and lymphangiogenesis [25,29-31]. Important for these physiological functions is the tight regulation of protein levels and an asymmetric distribution of ephrins and Eph receptors, for instance the asymmetrical arteriovenous expression of EB2 and EphB4 [23,26,32,33]. Eph-ephrin binding and clustering triggers a bi-directional signaling that is mediated by interactions of the cytoplasmic tails with cytosolic factors [30]. In EB2, activation of the signaling cascade depends on the C-terminal 33 amino acids, and EB2 knockout or truncation of just the catalytic cytoplasmic domain resulted in a signaling-defective EB2 which had lost its ability to promote vascular remodeling [34,35].

Since the expression levels of EB2 are highly regulated *in vivo *[23,32,33] and levels of viral entry receptors can be crucial for efficient virus entry and replication, the goal of this study was to determine if changes in EB2 receptor expression on the surface of NiV target cells influence NiV infection. We found that an overexpression of EB2 interfered with virus entry and NiV glycoprotein-mediated cell-to-cell fusion in F and G-transfected cells as well as in NiV-infected cells. Whereas the reduction in syncytia formation can be explained by the downregulation of the NiV glycoproteins from the cell surface, inhibition of virus entry is likely due to an oversupply of EB2 surface receptors interfering with efficient virus-cell fusion and subsequent NiV entry. Fusion assays and infection studies in cells expressing a tail-truncated and thus signaling-defective EB2 revealed that the catalytic cytoplasmic domain of EB2 is not involved in this process.

## Results

### Increasing surface expression levels of the NiV receptor EB2 interfere with NiV glycoprotein-mediated cell-to-cell fusion

**Figure 1:**
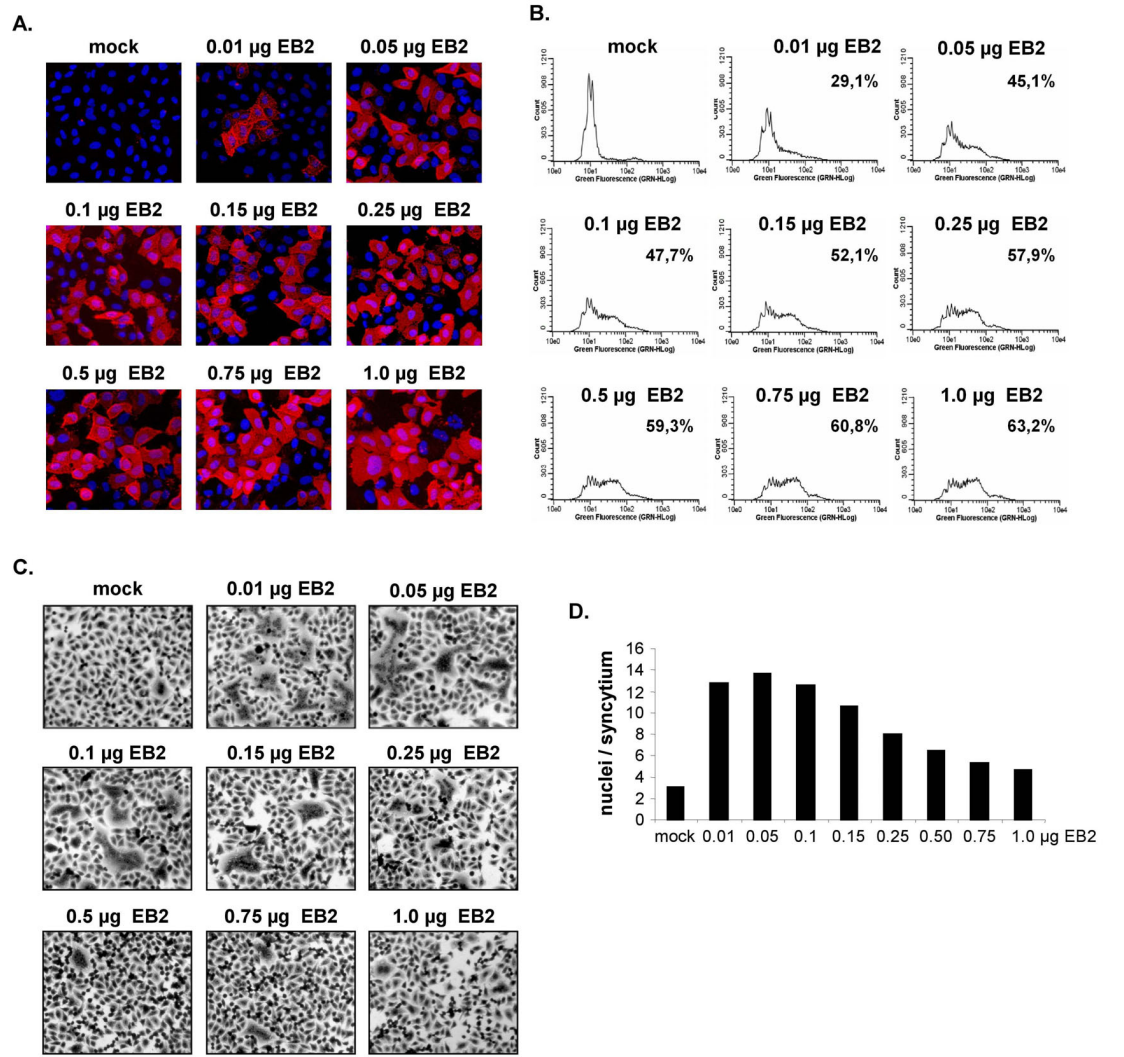
**EB2 surface expression and NiV glycoprotein-mediated cell-to-cell fusion in HeLa cells transfected with different amounts of pCAGGS-EB2**. (A) Receptor-negative HeLa cells were transfected with the indicated quantities of an EB2-encoding pCAGGS vector. At 24 h p.t., immunostaining was performed using recombinant EphB4/Fc and rhodamine-conjugated secondary antibodies. Nuclei were visualized by DAPI staining. (B) HeLa cells expressing different amounts of EB2 were incubated with an EB2-specific antibody followed by FITC-conjugated secondary antibodies. FACS analysis was performed at 24 h p.t. (C) HeLa cells were cotransfected with constant quantities of plasmids carrying the NiV F and G genes and the indicated amounts of pCAGGS-EB2. To visualize cell-to-cell fusion, cells were fixed and incubated with Giemsa staining solution at 24 h p.t.. Representative microscopic fields were photographed. (D) Syncytium formation of HeLa cells expressing different amounts of EB2 as shown in panel C was quantified by counting and averaging the number of nuclei per syncytium of twenty randomly chosen syncytia.

A correlation of expression levels of cell-surface receptors and infection efficiency has been shown for many different viruses. Increased receptor expression had either a beneficial effect on virus replication or had no effect [36-43]. To determine the influence of differences in receptor expression on NiV replication, we first analyzed the effect on glycoprotein-mediated fusion in the absence of a NiV infection. To monitor EB2 surface expression, EB2-negative HeLa cells were transfected with increasing amounts of EB2 plasmid DNA (pCAGGS-EB2), and were analyzed at 24 h post transfection (p.t.) by immunostaining (Fig. 1A) and FACS analysis (Fig. 1B). For immunofluorescence staining, living cells were incubated with recombinant EphB4/Fc. Surface-bound EphB4/Fc was then detected with a rhodamine-conjugated anti-human IgG antibody. FACS analysis of surface-expressed EB2 was performed using an EB2-specific antibody and FITC-conjugated anti-goat secondary antibodies. As shown in Fig. 1A and 1B, the number of EB2-positive cells raised with increasing quantities of EB2 DNA. In the sample transfected with 1 μg pCAGGS-EB2, 63.2% of the cells expressed EB2 on the cell surface. The mean fluorescence values, thus the mean receptor densities, were the same in all EB2-transfected cells indicating that cultures with more EB2-positive cells contained an increased total number of cells with higher EB2 expression levels, but maximal EB2 surface expression levels were not upregulated by transfection of more plasmid DNA.

To analyze the ability of cell cultures transfected with different EB2 plasmid concentrations to support NiV glycoprotein-mediated cell-to-cell fusion, constant and optimized ratios of NiV F and G protein encoding plasmids were cotransfected, and syncytia formation was monitored at 24 h p.t. by staining with Giemsa solution (Fig. 1C). Since HeLa cells do not express endogenous EB2, mock-transfected HeLa cells did not support any NiV glycoprotein-specific fusion (Fig. 1C, mock). As expected, EB2 transfection was required to render cells susceptible for cell-to-cell fusion. However, pronounced syncytia formation was only seen in cells transfected with low amounts of pCAGGS-EB2, higher amounts interfered with efficient cell-to-cell fusion. When we determined the mean size of syncytia by counting and averaging the number of nuclei per syncytium (Fig. 1D), we found the largest syncytia (14 nuclei) in HeLa cells transfected with only 0.05 μg pCAGGS-EB2. Then, the size decreased stepwise with increasing DNA amounts down to 4.9 nuclei per syncytium in cells transfected with 1 μg pCAGGS-EB2.

**Figure 2:**
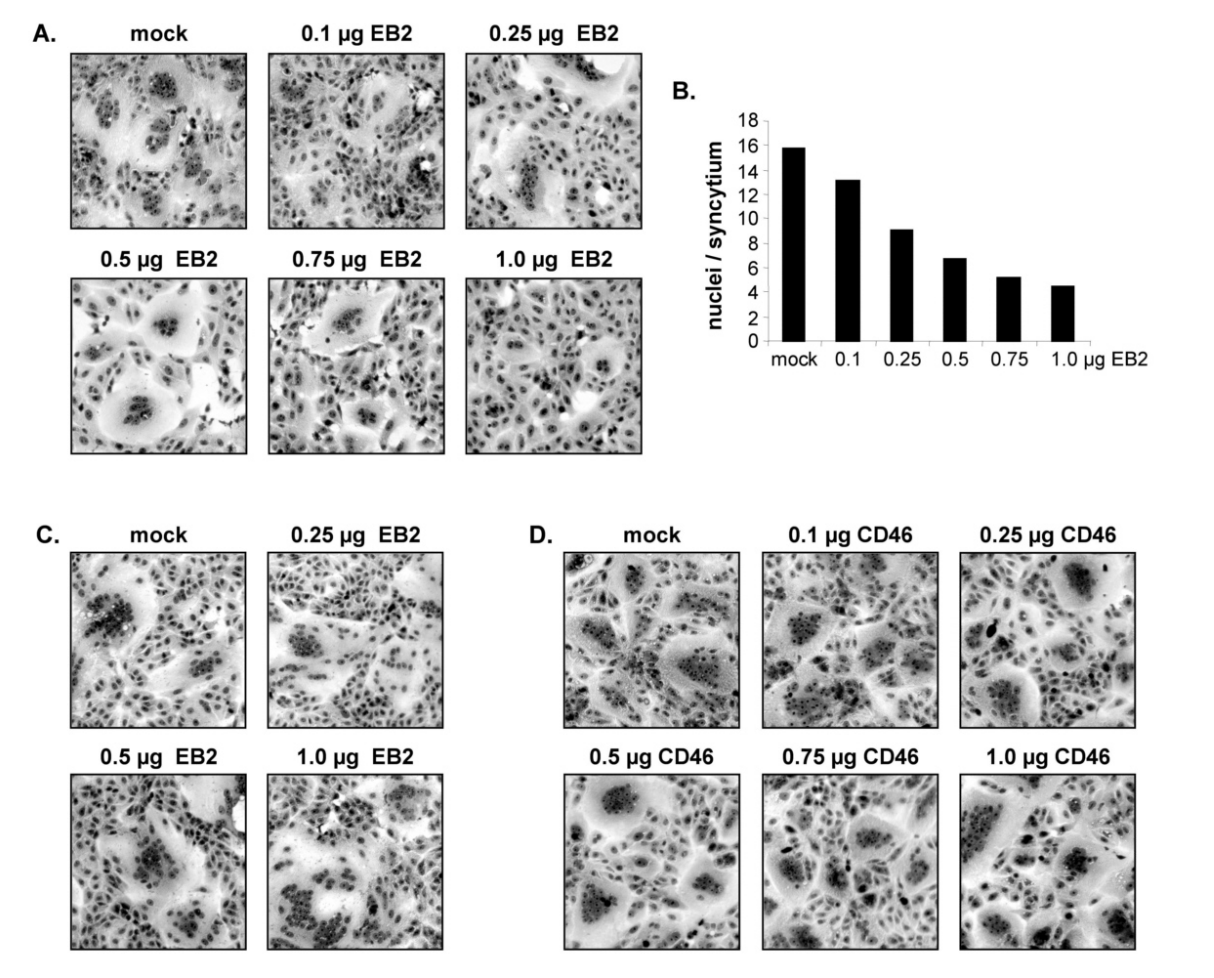
**NiV and MV glycoprotein-mediated cell-to-cell fusion in Vero cells overexpressing different amounts of EB2 or CD46**. (A) Vero cells were cotransfected with plasmids encoding the NiV F and G protein and increasing amounts of pCAGGS-EB2. Cell-to-cell fusion was visualized by Giemsa staining at 24 h p.t.. (B) Quantification of syncytium formation was performed as described in the legend to Fig. 1D. (C) Cells transfected with constant amounts of the MV glycoproteins and the indicated amounts of EB2 were Giemsa stained at 15 h p.t.. (D) Cells were transfected with constant amounts of plasmids encoding NiV F and G proteins and different amounts of plasmids encoding the CD46 gene. At 24 h p.t., syncytium formation was visualized by Giemsa staining.

To test if differences in EB2 expression also affect syncytium induction in NiV permissive cells expressing endogenous EB2, cotransfection of the NiV glycoprotein genes in addition to various amounts of pCAGGS-EB2 was performed in Vero cells. As anticipated, NiV F and G induced syncytium formation in mock-transfected cells (Fig. 2A, mock). Supplemental expression of exogenous EB2, even at the lowest concentration tested (0.1 μg DNA), resulted in decreased cell-to-cell fusion (Fig. 2A and 2B). In accordance with HeLa cells, EB2 overexpression in Vero cells clearly led to downregulation of NiV glycoprotein-induced syncytia formation. FACS analysis to quantify EB2 expression levels in transfected Vero cells again showed that with rising amounts of EB2 DNA an increasing percentage of Vero cells (up to 40% in cells transfected with 1 μg DNA) expressed EB2 at higher surface densities (data not shown). The mean fluorescence values in this cell population expressing additional plasmid-encoded EB2 was about 10-fold higher than in Vero cells expressing endogenous EB2 only (mock transfected cells). To exclude that these higher EB2 expression levels have a general downregulating effect on paramyxovirus cell-to-cell fusion, we investigated the effect of EB2 overexpression on syncytium formation caused by the measles virus Edmonston (MV_Edm_) glycoproteins F and H. MV_Edm _does not bind to EB2 but uses CD46 as entry receptor which is also endogenously expressed in Vero cells [44]. Cells were transfected with the MV_Edm _F and H genes together with different amounts of pCAGGS-EB2 and syncytia formation was analyzed at 15 h p.t.. In contrast to what we had observed for NiV, EB2 overexpression had no negative effect on MV_Edm _glycoprotein-mediated cell-to-cell fusion (Fig. 2C). This demonstrates that higher amounts of EB2 do not generally interfere with paramyxoviral glycoprotein-induced fusion but specifically inhibit NiV F- and G-mediated syncytia formation. When we expressed the NiV F and G protein in the presence of increasing amounts of CD46 we again did not see any effect on syncytia formation (Fig. 2D). Therefore, the downregulating effect on NiV glycoprotein-mediated fusion by EB2 is specific.

### EB2 overexpression can downregulate surface expression of the NiV glycoproteins

**Figure 3:**
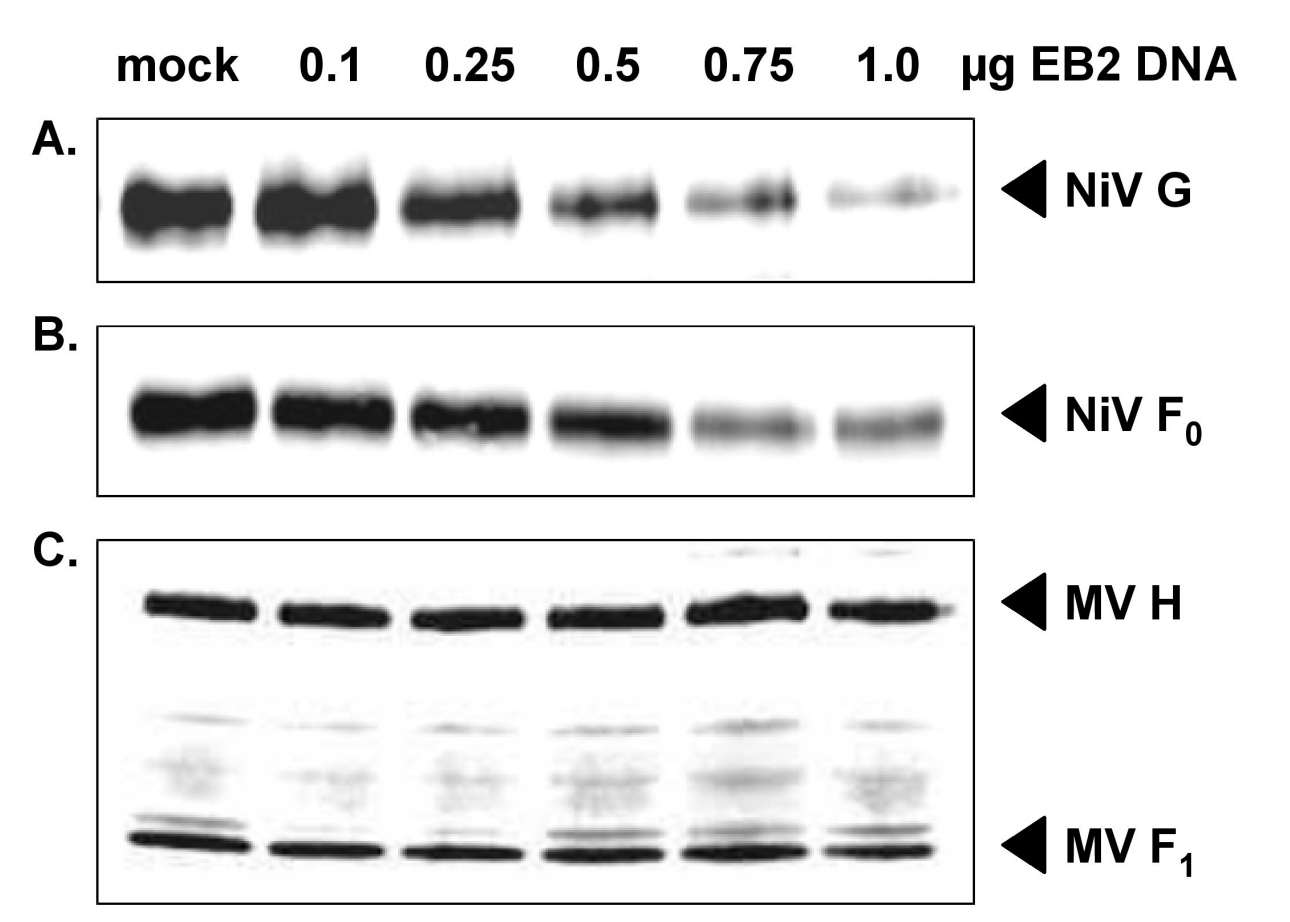
**Surface expression of NiV and MV glycoproteins in the presence of increasing amounts of EB2**. Vero cells were cotransfected with constant amounts of NiV F- or G-encoding plasmids and the indicated amounts of pCAGGS-EB2. At 24 h p.t., cells were surface biotinylated and lysed. (A) Immunoprecipitation of NiV G was carried out using a polyclonal NiV antiserum. After separation on a 12% SDS gel under reducing conditions and blotting to nitrocellulose, surface-biotinylated G proteins were detected by IRDye 800-conjugated streptavidin using a LiCor-Odyssey imager. (B) NiV F was immunoprecipitated with an F-specific antiserum, separated by SDS-PAGE under non-reducing conditions and further processed as described above. (C) Vero cells were cotransfected with constant amounts of plasmids encoding the MV F and H proteins and the different amounts of pCAGGS-EB2. Immunoprecipitation of the MV glycoproteins was carried out using F- and H-specific antibodies. After separation by SDS-PAGE under reducing conditions and blotting, proteins were detected as described above.

To determine if EB2 overexpression affects surface expression of the NiV F or G protein, Vero cells were cotransfected with constant amounts of plasmids encoding the NiV glycoproteins F and G in addition to varying amounts of pCAGGS-EB2. At 24 h p.t., cells were surface biotinylated followed by immunoprecipitation of F and G proteins. After separation by SDS-PAGE and blotting to nitrocellulose, surface expressed NiV glycoproteins were detected by IRDye 800-conjugated streptavidin. As shown in Fig. 3A and 3B, EB2 overexpression reduced the expression levels of F and G protein on the cell surface in a concentration-dependent manner. The finding that surface expression of the MV glycoproteins F and H was not influenced by EB2 transfection (Fig. 3C) clearly suggests that surface downregulation of NiV glycoprotein complexes is due to specific interactions with EB2.

### Increased EB2 surface expression interferes with productive NiV infection

**Figure 4:**
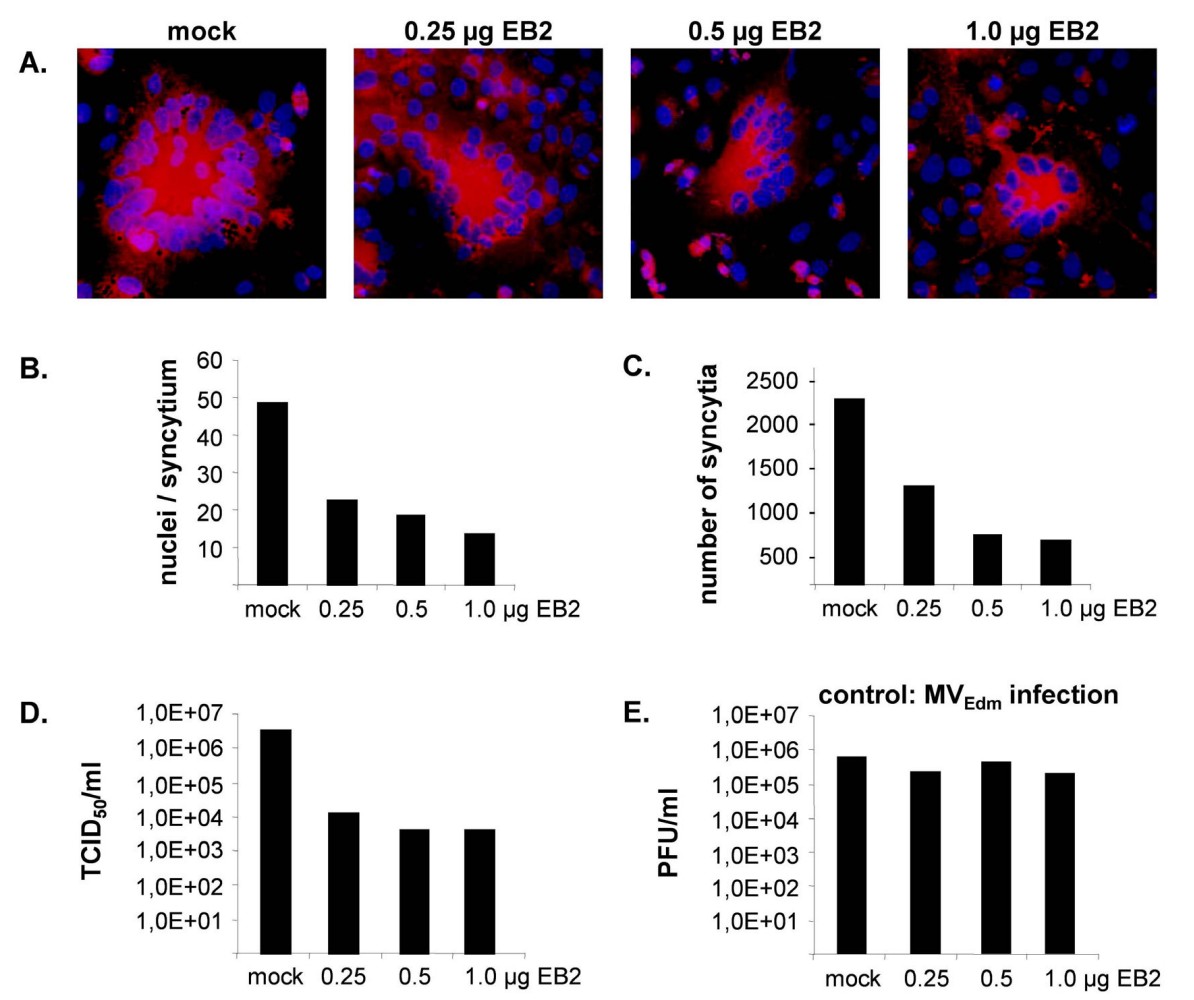
**Influence of EB2 overexpression on NiV and control MV_Edm _infection**. (A) Vero cells transfected with the indicated amounts of pCAGGS-EB2 were infected with NiV at a MOI of 1 at 15 h after transfection. At 24 h p.i., cells were fixed and an immunostaining was performed using a NiV-specific guinea pig antiserum and rhodamine-conjugated secondary antibodies. Nuclei were visualized by DAPI staining. (B) Nuclei per syncytium were quantified as described in the legend to Fig. 1D. (C) To determine the amount of initially NiV-infected cells, the total number of NiV-positive syncytia on each coverslip was counted. (D) Virus titers in the supernatant were determined by the TCID_50 _method at 24 h p.i.. (E) Vero cells transfected with different quantities of pCAGGS-EB2 were infected with MV_Edm _at a MOI of 1. Virus titers in the supernatant were determined by plaque assay at 42 h p.i..

To analyze the effects of additional EB2 expression in the context of a NiV infection, Vero cells were transfected with different amounts of the EB2-encoding plasmid, transferred to the BSL4 facility and subsequently infected with NiV at a multiplicity of infection (MOI) of 1. NiV-positive cells were stained by indirect immunofluorescence analysis at 24 h post infection (p.i.) to reveal the size and the number of syncytia. To quantify virus production, virus titers in the supernatant were determined by the TCID_50 _method. For immunostaining, NiV-infected cells were fixed and incubated with a NiV-specific guinea pig antiserum and rhodamine-conjugated secondary antibodies. Cell nuclei were counterstained with DAPI. Merged pictures of the rhodamine and DAPI fluorescence channels are shown in Fig. 4A and demonstrate that all Vero cell cultures transfected with different amounts of pCAGGS-EB2 could be infected with NiV. However, the number and size of syncytia appeared to be reduced in cells transfected with pCAGGS-EB2. This was confirmed by determining the average size of the NiV-induced syncytia (Fig. 4B). Whereas syncytia in mock-transfected cells contained about 50 nuclei in average, cells transfected with 0.25 μg, 0.5 μg or 1 μg of EB2 plasmid DNA produced syncytia with 22, 19 or 15 nuclei, respectively. To evaluate if differences in EB2 expression also affect virus entry, the total number of syncytia in each sample was counted and was also found to be reduced in EB2-transfected cells (Fig. 4C). Since one syncytium originates from one initially infected cell, this finding clearly indicates that not only NiV-mediated cell-to-cell fusion but also virus entry is impaired in Vero cells overexpressing EB2. In agreement with the decreased total number of infected cells and the less efficient spread via cell-to-cell fusion, the amount of infectious NiV particles released into the cell supernatant was also drastically diminished. Virus titers were reduced by more than 100-fold (Fig. 4D). To examine if inhibition of infection by EB2 overexpression is specific for NiV, control studies were performed with MV_Edm_. Vero cells transfected with various quantities of pCAGGS-EB2 were infected with MV_Edm _at a MOI of 1. Since no infectious virus could be detected at 24 h p.i., the amount of virus particles released from the cells was determined at 42 h p.i. by plaque assay. In contrast to NiV, MV_Edm _virus titers in the supernatant of mock- and EB2-transfected Vero cells were similar (Fig. 4E) demonstrating that MV_Edm _infection was not affected by variations in EB2 expression. We also analyzed MV_Edm _replication in the presence of increased levels of its own receptor CD46, but we did not observe any negative influence of CD46 overexpression on productive MV_Edm _infection (data not shown). We thus conclude that the negative effect of EB2 overexpression on productive virus replication is specific for NiV.

### Cytoplasmic-tail truncated EB2 also interferes with NiV-mediated cell-to-cell fusion and productive infection

**Figure 5:**
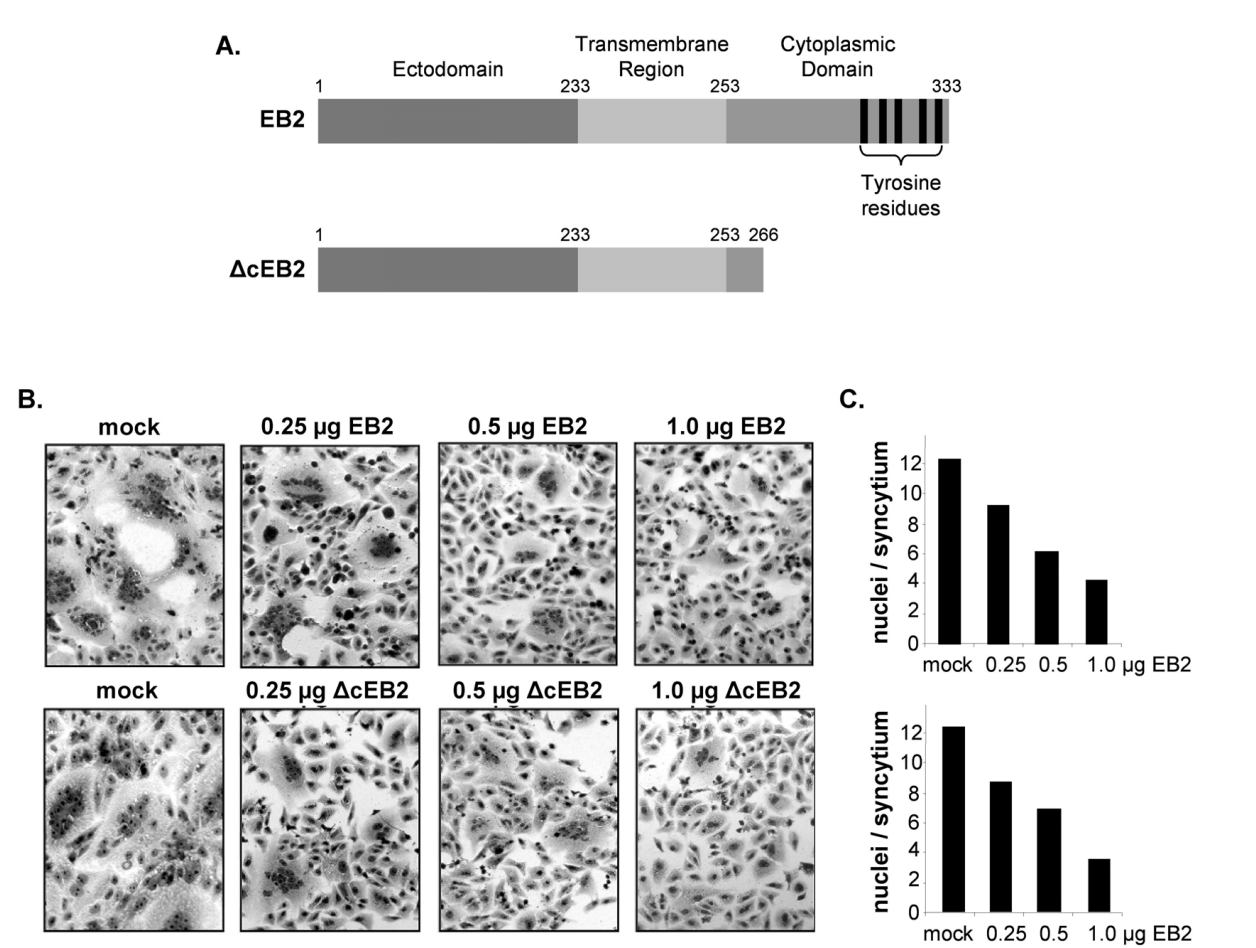
**Influence of EB2 and ΔcEB2 overexpression on NiV glycoprotein-mediated cell-to-cell fusion**. (A) Schematic diagram of EB2 and ΔcEB2. Numbers indicate the amino acid positions. (B) Vero cells were cotransfected with constant amounts of NiV glycoprotein DNA and the indicated quantities of plasmids encoding either EB2 or ΔcEB2 protein. After 24 h, cells were fixed and stained with Giemsa solution. (C) Quantification of syncytium formation was performed as described in the legend to Fig. 1D.

**Figure 6:**
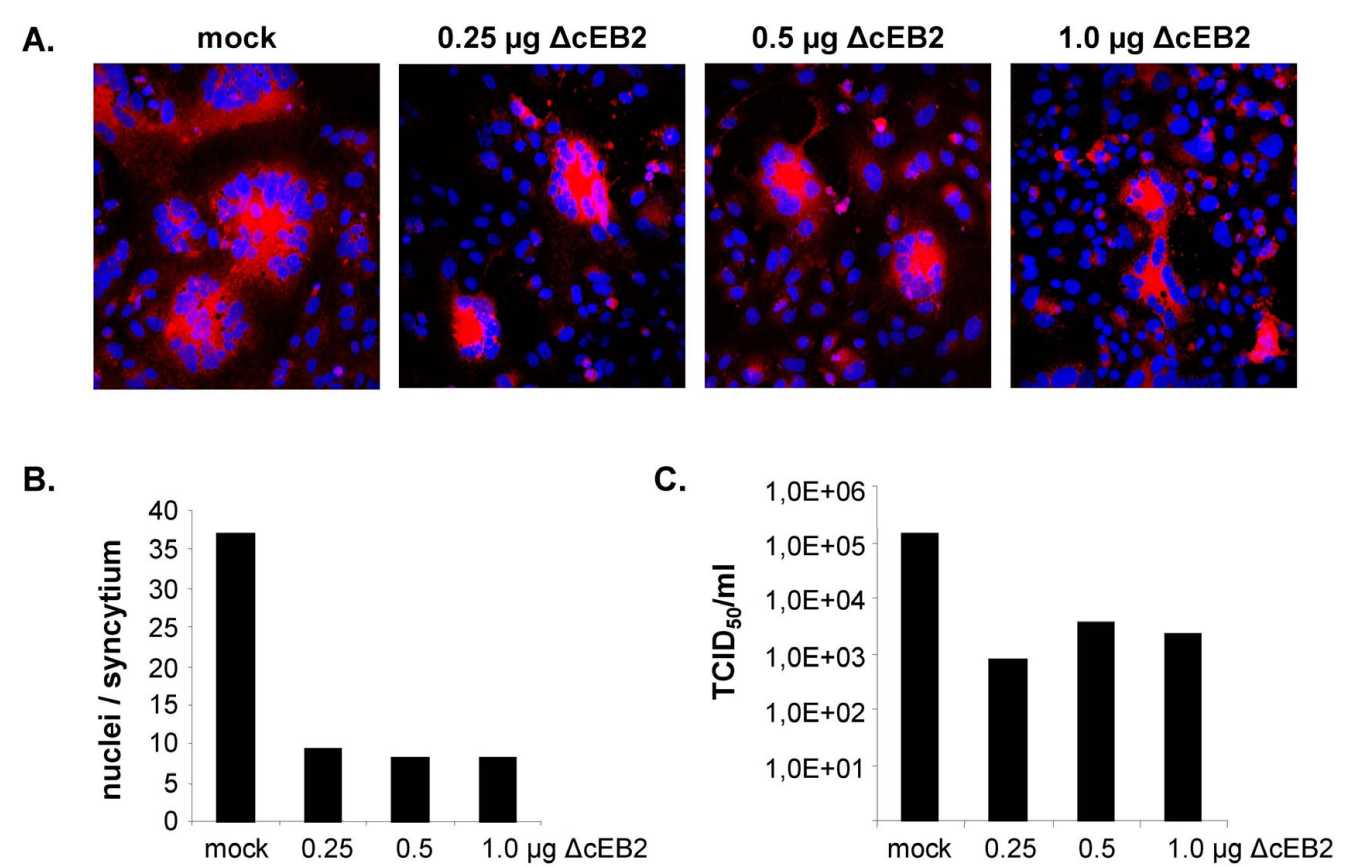
**Influence of increased ΔcEB2 expression levels on NiV infection**. At 24 h p.t., Vero cells transfected with the indicated amounts of pCAGGS-ΔcEB2 were infected with NiV at a MOI of 1. (A) At 15 h p.i., NiV-positive cells were stained as described in the legend to Fig. 4A. (B) Quantification of cell-to-cell fusion was carried out as described in the legend to Fig. 1D. (C) Cell free virus was determined by the TCID_50 _method.

Clustering of EB2 by NiV G protein binding during virus entry may be an essential component of these processes and might trigger EB2 signaling. Supporting this idea, it was recently shown that the critical residues in EB2 involved in interaction with NiV G are the same required for interaction with the EphB2 receptor [27]. After NiV G-induced EB2 clustering, NiV entry might be influenced by proteins interacting with the catalytic domain of the EB2 cytoplasmic tail, such as proteins containing PDZ domains which can stabilize high-ordered clustering into oligomeric arrays [45]. The density of this clustering or effects of EB2 signaling on actin cytoskeleton rearrangements may modulate the efficiency of virus-cell fusion. Therefore, the negative effect of EB2 overexpression on NiV entry could be the result of an overshooting EB2 signaling. To evaluate this idea, we decided to study the influence of a tail-truncated ΔcEB2 that lacks the C-terminal 67 amino acids on NiV replication [46] (Fig. 5A). First, we analyzed the effect of increased ΔcEB2 surface expression on NiV glycoprotein-mediated cell-to-cell fusion in Vero cells by transfecting the NiV F and G genes in addition to different amounts of EB2 or ΔcEB2 plasmid DNA. As with wildtype EB2, an expression level-dependent reduction of the size of syncytia was observed in ΔcEB2-expressing cells (Fig. 5B and 5C). To investigate the effect of increased amounts of tail-truncated EB2 on NiV infection, ΔcEB2-transfected Vero cells were infected with NiV and syncytium formation and virus production was monitored at different time points p.i.. The quantitative analysis at 15 h p.i. is depicted in Fig. 6 and shows that overexpression of tail-truncated EB2 affected productive NiV infection to similar extents as full-length EB2 (Fig. 4). Transfection of only 0.25 μg of ΔcEB2 plasmid DNA already drastically interfered with NiV-induced cell-to-cell spread (Fig. 6B) and reduced the release of infectious viruses by 2 to 3 log steps (Fig. 6C). Higher DNA concentrations had no further substantial effects. This indicates that receptor overexpression can downregulate productive NiV infection by interfering with virus entry and F- and G-mediated cell-to-cell fusion but cannot completely prevent infection.

### Cytoplasmic-tail truncated EB2 can function as NiV entry receptor

**Figure 7:**
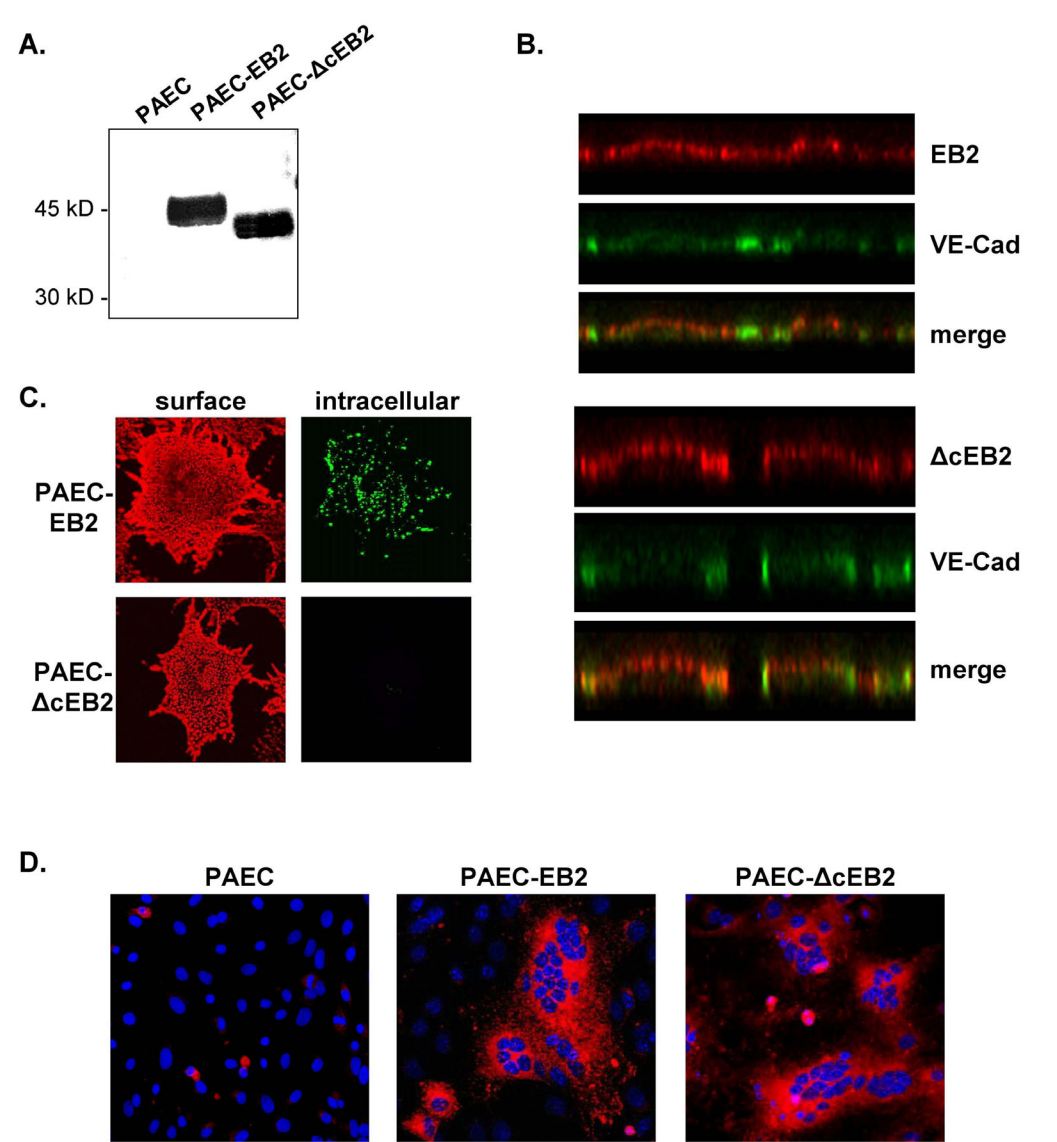
**Characterization and NiV infection of EB2- and ΔcEB2-expressing PAEC**. (A) EB2 and ΔcEB2 were immunoprecipitated from PAEC-EB2 and -ΔcEB2 cell lysates, separated on a SDS gel and analyzed by Western blot analysis using an EB2-specific antibody. (B) Stably EB2- and ΔcEB2-expressing PAEC were seeded on permeable filter supports. After 7 days, apical and basolateral surfaces were stained with EphB4/Fc and rhodamine-conjugated secondary antibodies. After fixation and permeabilization, cells were incubated with anti-VE-cadherin antibodies (VE-Cad) and FITC-conjugated secondary antibodies. (C) PAEC-EB2 and -ΔcEB2 were incubated with recombinant EphB4/Fc for 1 h at 37°C to allow binding and endocytosis to occur. Surface-remained EphB4/Fc was detected with rhodamine-conjugated secondary antibodies (surface) and internalized EphB4/Fc was stained after fixation and permeabilization by FITC-conjugated secondary antibodies (intracellular). (D) EB2- or ΔcEB2-expressing PAEC were infected with NiV at a MOI of 1. At 24 h p.i., immunostaining was performed as described in the legend to Fig. 4A.

The finding that overexpression of ΔcEB2 interfered with NiV infection suggests that tail-truncated EB2 can also function as NiV binding partner. To directly test if ΔcEB2 can be used as NiV entry receptor or if it even provides a more effective receptor than full-length EB2 because it no longer functions in signaling, we analyzed NiV infection in HeLa cells and porcine aortic endothelial cells (PAEC) stably expressing either wildtype or a tail-truncated ΔcEB2. As we got similar results for both cell lines, only the results obtained for the PAEC, a well-studied cell line in terms of EB2 functions [46] are shown. To control the protein expression, EB2 and ΔcEB2 proteins were immunoprecipitated from cell lysates and subjected to western blot analysis using an EB2-specific antibody. As shown in Fig. 7A, expression of EB2 and ΔcEB2 in stably transfected PAEC is comparable. To characterize surface localization of EB2 and the tail-truncated variant, double-labeling experiments for EB2 and VE-cadherin, a marker protein for adherens junctions in endothelial cells, were performed. PAEC-EB2 and -ΔcEB2 were seeded on porous filter membranes and cultured for 7 days to form a polarized endothelial cell monolayer. Surface-expressed EB2 and ΔcEB2 was detected by incubation with EphB4/Fc on ice and subsequent treatment with rhodamine-conjugated anti-human IgG antibodies. Then, cells were permeabilized and stained with a VE-cadherin antibody and a FITC-conjugated secondary antibody. Analysis of vertical sections through the endothelial cell monolayers identified an equal luminal expression of both EB2 and ΔcEB2 (Fig. 7B). To control the loss of function of the tail-truncated EB2, an EphB4 receptor body uptake experiment was performed [33]. For this, PAEC-EB2 and PAEC-ΔcEB2 were incubated with recombinant EphB4/Fc for 1 h at 37°C to allow binding of EphB4/Fc and co-endocytosis of EB2 and EphB4/Fc to occur. Surface-remained EphB4/Fc was visualized by incubation with rhodamine-conjugated secondary antibodies at 4°C. After fixation and permeabilization, intracellular EphB4/Fc was stained with FITC-conjugated secondary antibodies. In PAEC stably expressing wildtype EB2, numerous fluorescent intracellular vesicles (green dots) were found (Fig. 7C, PAEC-EB2). In contrast, in PAEC-ΔcEB2 expressing cells, no intracellular complexes were detected demonstrating that tail-truncated EB2 is no longer endocytosed. To analyze the receptor function of tail-truncated EB2, PAEC-EB2 or PAEC-ΔcEB2 were infected with NiV at a MOI of 1. To monitor the infection at 24 h p.i., NiV-positive cells and syncytia were detected by immunostaining and virus titers in the supernatant were quantified by the TCID_50 _method. As shown in Fig. 7D, extensive cell-to-cell fusion could be detected in both PAEC-EB2 and PAEC- ΔcEB2. The average size as well as the number of syncytia was similar. Accordingly, the amount of infectious virus particles released into the supernatant was the same (3.1 × 10^6 ^TCID_50_/ml for both cell lines). The finding that there are no substantial differences in the amount of initially infected cells, NiV-mediated cell-to-cell fusion and virus titers demonstrates that the tail-truncated ΔcEB2 can fully function as NiV entry receptor in endothelial cells, the predominant target cells of the NiV infection *in vivo*.

## Discussion

The major and new finding of this study is that productive NiV replication is counteracted by overexpression of the main NiV receptor EB2. By transfection of different amounts of EB2 gene into EB2-negative HeLa or EB2-positive Vero cells and quantification of syncytia formation upon coexpression of the two NiV envelope glycoproteins and in NiV-infected cells, we found that increasing amounts of EB2 plasmid DNA augmented the total number of cells expressing high levels of EB2 on the surface without changing the maximal EB2 surface density. Unexpectedly, EB2 expression higher than a very low threshold in HeLa cells or any additional EB2 expression in Vero cells resulted in a decreased cell-to-cell fusion, and also interfered with efficient virus entry. This negative effect on syncytia formation is mostly due to the fact that the expression levels of the NiV glycoproteins on the cell surface were reduced. However glycoprotein downregulation cannot be the reason for the reduced virus entry. To assess if the negative effect of elevated EB2 expression levels on virus entry and cell-to-cell fusion is linked to direct or indirect effects of an increased EB2 signaling, a cytoplasmic tail truncated and therefore signaling-defective EB2 (ΔcEB2) was expressed in Vero cells. Interestingly, overexpression of ΔcEB2 had a similar negative effect on virus entry and fusion, clearly showing that the detrimental influence of elevated receptor expression on NiV infection is not linked to EB2 signaling. NiV infection of endothelial cells expressing either full-length or truncated EB2 was similar in terms of virus entry and replication finally confirming that the receptor function of EB2 is independent of its cytoplasmic tail.

For their multiple physiological functions in angiogenesis, axon guidance, cell migration and neovascularization of tumors [47,48], ephrins and Eph receptors must cluster to trigger bi-directional signaling into the ephrin-expressing cell (reverse signaling) and the contacting Eph receptor expressing cell (forward signaling) [49]. EB2-mediated reverse signaling is known to depend on a catalytic domain comprising the conserved last 33 residues of the cytoplasmic domain which contains a PDZ domain binding motif and five conserved tyrosine residues [30,50,51]. One of the two known signaling pathways activated by B ephrins depends on the phosphorylation of the tyrosine residues and subsequent binding of Src-homology-2-domain-containing adaptor molecules like Grb4 initiating a cascade of signaling events that regulate cytoskeleton dynamics [52]. The second signaling pathway depends on the interaction with PDZ domain binding proteins like Grip1, Grip2, syntenin, Par3, PICK1 or Dvl2 inducing cluster formation, or binding partners with PDZ domains linked to a functional unit, for instance PTP-BL, Tiam1 or PDZ-RGS3 regulating G protein-coupled signaling events, finally resulting in guided cell migration [25,30,53]. In a previous report, the interesting idea was proposed that proteins containing a PDZ domain or other proteins which can interact with the EB2 cytoplasmic tail, may influence NiV entry into host cells and therefore could be a potential target for therapeutic treatments [54]. This concept that putative signals sent through EB2 upon interaction with NiV G may be an essential component of the entry process was mainly based on the finding that the NiV-G protein is a tetramer as it is also proposed for Eph receptors [28,55,56]. Thus, G binding might lead to EB2 clustering and activation of the signaling cascade. However, the data presented in this paper indicate that cellular binding partners of the EB2 catalytic domain are not involved in NiV entry, because a tail-truncated EB2 fully functions as host cell receptor and downregulates NiV infection when it is overexpressed. But even if EB2-mediated signaling per se is obviously not involved in virus entry and spread via cell-to-cell fusion, it remains to be determined if NiV binding to its receptor triggers EB2-mediated signaling possibly affecting host cell functions or apoptosis, as it is described for HIV [57,58].

It is well known that expression levels of viral entry receptors can be crucial for virus infection. For instance, adenovirus binding to the coxsackievirus-adenovirus receptor (CAR) and subsequent infection clearly decreased if CAR expression is reduced after cytokine treatment [42]. Similarly, the density of cell surface-expressed CD81 was shown to be a key determinant for productive hepatitis C virus (HCV) entry into host cells. Cell susceptibility to HCV infection could be increased by augmenting CD81 surface densities up to a certain threshold, further increase did not have any additional positive or negative effects [37]. In the case of the human immunodeficiency virus (HIV) cell surface concentrations of receptors and coreceptors also control infection efficiency. Concentrations of CD4 and CCR5 required for efficient infections by HIV were found to be interdependent, requirements for each were increased when the other component was limited [40]. For several other retroviruses positive correlations of receptor overexpression and virus infection were also reported [36,38,39,41,43]. In all reports so far, upregulation of receptor densities had either no or beneficial effects on virus replication. A negative effect of increased virus receptor expression on virus entry and productive infection as demonstrated in this study has never been reported, and thus might reflect a unique characteristic of the highly virulent NiV.

Coexpression of virus receptors and viral receptor binding glycoproteins in one cell can result in complex formation and retention of both proteins in intracellular compartments leading to downregulation of the receptor on the cell surface. This phenomenon of receptor interference is described for several viral proteins which are known to interact with or even induce degradation of their cellular receptors [59-65]. Even though Sawatsky et al. [66] described EB2 surface expression to be unchanged after coexpression of NiV G, our finding that the NiV glycoprotein surface expression levels are downregulated in EB2 expressing cells indicates an intracellular interaction and subsequent retention of the two proteins. However, this effect that leads to reduced cell-to-cell fusion efficiency does not explain the less efficient virus entry into Vero cells expressing additional EB2. Reduced infection of these cultures is most likely due to an imbalance between cellular receptors and viral receptor binding proteins in the virus envelope. Since fusion and the lateral mobility of paramyxoviral glycoproteins in the target cell membrane correlate [67], NiV G interactions with too many receptor molecules on a host cell likely hinders the virus-cell fusion process by interfering with the optimal formation or mobility of fusion pore complexes required for virus-cell fusion and subsequent virus entry. Our observation that cell-to-cell fusion is decreased if cells expressing the NiV glycoproteins were mixed with cells expressing increased amounts of EB2 supports the model that the balance between the amount of fusogenic glycoproteins on one membrane and the density of receptors on adjacent membranes critically determines fusion efficiency (Thiel, unpublished observations). Given that more cells in a culture express too high EB2 concentrations, more viral NiV G proteins are clustered with receptors. This prevents or slows down the fusion process required for virus entry or cell-to-cell fusion.

Since high viral replication in the central nervous system is likely an important factor for the high mortality rates of NiV infection in humans [14], EB2 expression levels may critically influence viral replication by regulating virus entry, virus spread by cell-to-cell fusion and particle release. Thus, fatal or nonfatal outcome of a NiV encephalitis might depend on EB2 expression levels. The histopathological finding that in brain tissues of NiV-infected patients, syncytial cells were only found in 27% of the cases whereas vasculitis, thrombosis and necrosis were seen in over 80% [13] might reflect differences in the EB2 expression levels resulting in different extents of NiV-mediated cell-to-cell fusion. Besides its possible influence on the outcome of the acute infection, differences in EB2 expression levels might also contribute to the relapse encephalitis which was found in some patients at 13–39 days after mild or asymptomatic acute NiV infection [12]. As it is well known, that EB2 expression *in vivo *is regulated by the local microenvironment within the vascular tree [33], and up-regulation of EB2 is controlled by the Notch pathway as well as by hemodynamic factors or vascular endothelial growth factor (VEGF) [33,68-70], NiV infection of brain endothelia might induce changes in the local microenvironment thereby inducing up- or downregulation of EB2 and influencing further virus spread. Using a suitable animal model [7,71,72], it remains to be elucidated if up- or downregulation of the NiV receptor is able to influence NiV spread *in vivo *and thus might be a potential therapeutic option for treatment of NiV encephalitis in early stages of infection.

## Conclusion

In summary, this paper demonstrates for the first time that overexpression of a virus receptor substantially interferes with virus infection on the level of virus entry and cell-to-cell spread by two independent pathways. Whereas cell-to-cell fusion is mainly reduced as a consequence of NiV glycoprotein downregulation from the cell surface, virus entry is rather impaired by disturbing the optimal balance between the amount of fusogenic glycoproteins in the virus envelope and receptors on the host cell surface.

## Methods

### Cell cultures

Vero (African green monkey kidney) and HeLa (human cervical cancer) cells were maintained in Dulbecco's modified minimal essential medium (DMEM, Gibco) containing 10% fetal calf serum (FCS), 4 mM L-glutamine, 100 units/ml penicillin and 0.1 mg/ml streptomycin. PAE (porcine aortic endothelial) cells were cultured in DMEM/F12 + GLUTAMAX (Gibco) supplemented with 10% FCS, penicillin and streptomycin.

### Virus infections

The NiV strain used in this study was isolated from human brain tissue (kindly provided by J. Cardosa) and propagated as described earlier [73]. For NiV infection, confluent cell monolayers of different cell lines were infected at a multiplicity of infection (MOI) of 1. After incubation for 1 h at 37°C, inocula were removed, cells were washed twice and then cultured with medium containing 2% FCS at 37°C. All work with live NiV was performed under BSL4 conditions.

Measles virus vaccine strain Edmonston (MV_Edm_) was grown and propagated on Vero cells as described previously [74]. For MV_Edm _infection studies, cells were infected with MV_Edm _at a MOI of 1. After incubation for 2 h at 37°C, virus was removed by extensive washings and cells were incubated with medium containing 2% FCS at 33°C.

### Plasmids and transfections

The NiV F and G glycoprotein open reading frames (GenBank™ accession number AF212302) were subcloned into the pczCFG5 expression vector as described by Moll *et al*. [75]. Cloning of the MV_Edm _glycoprotein genes (F and H) into the pCG expression vector has been described earlier [76]. The human EB2 gene and a C-terminally truncated EB2 version (encoding amino acid residues 1–266), designated as ΔcEB2 [46], were subcloned into the NotI site and SacI site of a pCAGGS expression vector [77]. Human CD46 gene was cloned into the pHßAPr.1-neo expression vector as specified previously [78].

Transfections of Vero and HeLa cells were performed by using the cationic liposome based transfection reagent Lipofectamine 2000 (Invitrogen) according to the supplier's instructions. Stably EB2- and ΔcEB2-expressing PAE cells were constructed as described by Füller *et al*. [46].

### EB2 surface staining

HeLa cells transiently expressing varying amounts of EB2 were cultured on coverslips. At 24 h post transfection (p.t.), recombinant mouse EphB4/Fc, a soluble EB2 receptor fused to the Fc region of human IgG (R&D Systems) was added at a concentration of 2 μg/ml for 1 h at 4°C. Bound EphB4/Fc was stained with rhodamine-conjugated anti-human IgG antibodies on ice (dilution 1:200; Jackson ImmunoResearch). Nuclei were visualized by 4',6-diamidino-2-phenylindole (DAPI) staining. Representative merged images of the DAPI and rhodamine channels were recorded with a Zeiss Axiovert 200 M microscope.

Stably EB2- and ΔcEB2-expressing PAE cells were cultured on 0,4 μm-pore size ThinCert polyethylenterephthalat filter supports (Greiner Bio-one). After 7 days, the apical and basolateral surfaces were incubated with recombinant EphB4/Fc (2 μg/ml; 2 h at 4°C) and rhodamine-conjugated secondary antibodies (dilution 1:200; 2 h at 4°C). To visualize the adherens junctions, cells were fixed with 4% paraformaldehyde (PFA), permeabilized with 0.1% Triton X-100 for 15 min and subsequently treated with anti VE-cadherin antibodies (Santa Cruz) at a dilution of 1:100 for 2 h at 4°C and fluorescein isothiocyanate (FITC)-conjugated anti-mouse IgG antibodies (dilution 1:200; 2 h at 4°C; Jackson ImmunoResearch). Filter membranes were analyzed with a confocal laser scanning microscope (LSM 510, Zeiss).

### Flow cytometry

HeLa or Vero cells were transfected with different amounts of EB2 DNA. At 24 h p.t., cells were detached with phosphate buffered saline (PBS) containing 5 mM EDTA, washed twice and 5 × 10^5 ^cells were subsequently incubated with an EB2-specific antibody (dilution 1:10; R&D Systems) for 45 min at 4°C. Primary antibodies were detected by FITC-conjugated anti-goat IgG secondary antibodies (dilution 1:100; Jackson ImmunoResearch) and flow-cytometric analyses were carried out with a FACScan (Guava Technologies). Since it has been shown that coexpression of NiV G protein did not influence the expression of EB2 on the cell surface [66], FACS analysis was performed in cells expressing EB2 only.

### Fusion assays

As it has been reported for the closely related HeV glycoproteins that the ratio of F to G plasmids transfected into cells can influence the efficiency of membrane fusion [79], we first tested the capacity of varying levels of pczCFG5-F and pczCFG5-G transfected into Vero cells to mediate cell-to-cell fusion. In agreement with what has been shown for the HeV glycoproteins, increased fusion was observed with greater amounts of the G protein plasmid. Since NiV glycoprotein-mediated syncytia formation was found to be maximal at a 1:5 ratio of NiV F to NiV G plasmid DNA, cells were cotransfected with constant amounts of plasmids encoding either the NiV glycoproteins F and G at this ratio or the MV glycoproteins F and H (optimal ratio of 1:1), in addition to varying amounts of pCAGGS-EB2, pCAGGS-ΔcEB2 or pHßAPr.1-neo-CD46, respectively. At 24 h (NiV) or 15 h (MV) p.t., cells were fixed with ethanol for 10 min and incubated with a 1:10 diluted Giemsa staining solution for 30 min to visualize syncytium formation. Representative microscopic fields were photographed. To quantify the size of syncytia, the number of nuclei per syncytium of twenty randomly chosen syncytia were counted and averaged.

### Surface biotinylation

Cell surface proteins were labeled with sulfosuccinimidobiotin (S-NHS-biotin; Pierce) and subsequently lysed in radioimmunoprecipitation assay buffer (RIPA) as described previously [17]. Immunoprecipitation of NiV F from surface-biotinylated cells was carried out with an F-specific antibody directed against amino acids 523 to 541 of the NiV F cytoplasmic domain (dilution 1:100; ImmunoGlobe Antikörpertechnik), and NiV G was immunoprecipitated with a polyclonal NiV-specific antiserum (dilution 1:350). MV glycoproteins were isolated using F- or H-specific monoclonal antibodies [76]. Immunoprecipitates were separated on a 12% polyacrylamide gel under reducing (NiV G, MV F and H) or non-reducing conditions (NiV F) and blotted to nitrocellulose. Nonspecific binding sites were blocked with 5% nonfat dry milk in PBS. To detect surface-biotinylated NiV and MV glycoproteins, blots were incubated with IRDye 800-conjugated streptavidin for 45 min at 4°C (Rockland; dilution 1:8000). Fluorescent signals were analyzed using a LiCor-Odyssey infrared imaging system (LI-COR Biosciences GmbH).

### NiV infection of cells expressing varying amounts of EB2 or ΔcEB2

Vero cells grown on coverslips were transfected with varying amounts of pCAGGS-EB2 or -ΔcEB2. At 15 h p.t., infection with NiV was performed as described above. NiV-positive cells or syncytia were detected by indirect immunofluorescence as described recently [15]. Briefly, after fixation with 4% PFA for 48 h, cells were permeabilized with methanol-acetone and incubated with a NiV-specific guinea pig antiserum (dilution 1:1000) for 1 h at 4°C. Primary antibodies were detected with rhodamine-conjugated anti-guinea pig IgG antibodies (Jackson ImmunoResearch; dilution 1:200; 45 min at 4°C). Nuclei were counterstained by DAPI. Images were recorded using a Zeiss Axiovert 200 M microscope.

The size of NiV-induced syncytia was quantified as described above. The number of initially infected cells in each sample was determined by counting the number of NiV-positive syncytia. To quantify virus release, virus titers in the supernatant were calculated by the 50% tissue culture infective dose (TCID_50_) method on Vero cells [80].

### Immunoprecipitation and Western Blot analysis of EB2 and ΔcEB2

Stably EB2- and ΔcEB2-expressing PAEC were lysed in immunoprecipitation buffer (1% Triton X-100, 0.15 M NaCl, 1 mM EDTA, 10 mM Tris-HCl, pH 7,4). EB2- and ΔcEB2 were immunoprecipitated with EphB4/Fc (dilution 1:100) and 100 μl of a suspension of protein A/G sepharose CL-4B (Pierce). After three washes, immunocomplexes were suspended in reducing sample buffer. Precipitates were subsequently separated on a 10% polyacrylamide gel and transferred to nitrocellulose. The blot was probed with 0,3 μg anti-EB2 (R&D Systems) followed by incubation with peroxidase-conjugated anti-goat IgG antibodies (dilution 1:4000; Jackson ImmunoResearch). EB2 and ΔcEB2 proteins were visualized with the enhanced chemoluminescence system (SuperSignal^® ^West Pico Chemoluminescent Substrate; Pierce) by exposure to an autoradiography film (GE Healthcare).

### Endocytosis of EB2 and ΔcEB2

Stably EB2- and ΔcEB2-expressing PAEC were seeded on coverslips and grown to subconfluency. Cells were then incubated with 2 μg/ml recombinant EphB4/Fc for 1 h at 37°C to allow binding and endocytosis to proceed. Surface-remained EphB4/Fc was stained with rhodamine-conjugated anti-human IgG antibodies (dilution 1:50) for 90 min at 4°C. After fixation and permeabilization with methanol-acetone, internalized EphB4/Fc was detected by FITC-conjugated anti-human IgG antibodies (dilution 1:500, Jackson ImmunoResearch) for 35 min at 4°C. Images of representative cells were recorded using a Zeiss Axiovert 200 M microscope.

## Competing interests

The authors declare that they have no competing interests.

## Authors' contributions

LT and SE carried out most of the experiments and helped to draft the manuscript. SD performed all work under BSL-4 conditions. AM designed the study. DP and HGA provided critical reagents. AM, DP and HA helped with the analysis and the interpretation of the data and drafted the manuscript. All authors read and approved the final manuscript.
